# Biomarkers of Oxidative Stress in Experimental Models and Human Studies with Nutraceuticals: Measurement, Interpretation, and Significance

**DOI:** 10.1155/2016/6159810

**Published:** 2016-01-27

**Authors:** Ilaria Peluso, Maura Palmery, Jara Pérez-Jiménez, Gregor Drummen

**Affiliations:** ^1^Center of Nutrition, Council for Agricultural Research and Economics (CREA-NUT), Via Ardeatina 546, 00178 Rome, Italy; ^2^Department of Physiology and Pharmacology “V. Erspamer”, “Sapienza” University of Rome, Piazzale Aldo Moro 5, 00185 Rome, Italy; ^3^Institute of Food Science, Technology and Nutrition, Spanish Research Council (ICTAN-CSIC), José Antonio Novais 10, 28040 Madrid, Spain; ^4^Cellular Stress and Ageing Program, Renal Pathobiology Program, Bio & Nano-Solutions, Enniskillener Straße 70, 33647 Bielefeld, Germany

Oxidative stress, the imbalance between reactive oxygen species (ROS) formation and enzymatic and nonenzymatic antioxidants, is involved in the pathogenesis and progression of many malignant, degenerative, and ageing-associated diseases. Most importantly, the chronic low grade inflammation associated with metabolic syndrome is characterized by a chronic oxidative stress condition and increased levels of cytokines. T. Magrone et al. investigated if polyphenol supplementation of farmed sea bass (*Dicentrarchus labrax L*.) would be a viable way to counteract the chronic inflammation present in farmed fish. The authors observed that, in supplemented fish, interleukins 1*β* and 6 were significantly decreased, whereas splenic interferon *γ* levels were increased. Concomitantly, a reduction in the number of spleen macrophages compared with control fish was detected. These results open up new perspectives for the use of antioxidants in farmed fish and might contribute to a more sustainable aqua culture.

Inflammation and oxidative stress are also induced by various exogenous agents and are therefore major components that not only determine the toxicity of such agents in somatic cells but also potentially affect the teratogenicity of reproductive cells. S. AE Bashandy et al. reported that increased arsenic levels significantly increased testicular malondialdehyde (MDA), tumour necrosis factor alpha (TNF-*α*), nitric oxide (NO), and sperm abnormalities and concurrently induced a decrease in testicular superoxide dismutase (SOD), catalase (CAT), reduced glutathione (GSH), and zinc levels. Furthermore, their results suggests that* Spirulina platensis* preparations may represent a potential therapeutic option to protect testicular tissue from arsenic toxicity. In particular, the improved antioxidant status of testicular tissues as a result of* Spirulina platensis* treatment can be deduced from the determined elevated levels of testicular SOD, CAT, zinc, and GSH and from the decrease in MDA in the* Spirulina platensis* + arsenic group as compared to the arsenic group.

In the study by S. A. Sheweita et al., the extracts of the medicinal plant* Alhagi Maurorum* (camel thorn plant) improved glucose levels, the lipid profile, and hepatic function in a rat model of diabetes. Moreover, treatment of diabetic rats with* Alhagi Maurorum* extracts improved the antioxidant status of hepatic tissues by increasing GSH levels and the activities of SOD, glutathione peroxidases (GPx), and glutathione transferase (GST) compared with the control group.

Next to their direct antioxidative properties, polyphenols can act as oxidants in some conditions, inducing redox-sensitive genes such as antioxidant enzymes. J. Marinic and coworkers found in a model of liver resection and intraperitoneal therapy that the higher liver regeneration induced by olive oil polyphenol extracts was preceded by an initial decrease of GSH and an increase of thiobarbituric acid reactive substances during the first 3 hours after hepatectomy, associated with the induction of GST, SOD, and CAT.

In the defence against ROS, both enzymatic and nonenzymatic antioxidants produced by cells and the low molecular weight molecules absorbed from food stuffs play vital roles in maintaining cellular homeostasis. S. Brundu and collaborators focused their study on the development of a reversed-phase HPLC method for the analysis of cysteine and reduced glutathione in biological samples from mice. Given the established and pivotal role of glutathione in many cellular functions, its determination in the assessment of oxidative stress is quite relevant. In particular, the authors validated their method in samples of diverse tissue origin, including spleen, lymph node, brain, and pancreas according to strict US and European standards.

Despite the fact that an increasing body of evidence suggests that natural functional compounds have bioremedial properties and might consequently be beneficial to human health, clinical evidence remains turbid. Since bioavailability is a key factor that determines if a component is taken up and transported to tissues and cells, not surprisingly, a significant research effort focuses on determining compound bioavailability in humans. X. Guo et al. reported results from a 5-year study that was conducted within the framework of the PREDIMED trial on subjects with high cardiovascular risk. In this study urinary total polyphenol excretion (TPE) was inversely associated with a number of clinical cardiovascular risk factors, namely, plasma glucose and triglycerides concentrations and systolic blood pressure. These results suggest that TPE could be useful as a marker of compliance in intervention studies with foods with high-polyphenol content. On the other hand, polyphenols can also exert their bioactivity without reaching the systemic circulation. Such poorly absorbable antioxidants primarily exert their protective effect against oxidative stress at the interface between the gastrointestinal lumen and gastrointestinal lining. As such, these antioxidants might be attractive therapeutics against oxidative damage-induced gastrointestinal diseases. E. Azzini and collaborators studied the effect of polyphenol-rich extracts from* Cichorium intybus* L. on Caco-2 cells in an* in vitro* monolayers model. Their results show that, at low concentrations, the polyphenol fractions indeed show antioxidative effects, but at higher concentrations they rapidly become cytotoxic. This effect is characterized by increased tight-junction permeability, epithelium abnormalities, and loss of plasma membrane integrity. The authors further confirm that the protective effect by polyphenol extracts from chicories occurs via the interaction with the mucopolysaccharide complexes in the glycocalyx.

Oxidative stress markers are important tools to assess both the redox status of subjects and the health-enhancing effects of antioxidants in humans. However, there is a lack of consensus, analytical validation, standardization, and reproducibility regarding many oxidative stress markers, evaluation models, and antioxidant assays. I. Peluso et al. investigated the relationship between the Peroxidation of Leukocytes Index Ratio (PLIR) and the increasingly studied postprandial metabolic stress. For this, in a pilot clinical trial with a functional cookie containing dark chocolate and pre- and probiotics, they measured PLIR in lymphocyte, monocytes, and granulocytes. The results of the investigation show that the determination of this parameter in healthy subjects may present some limitations during the postprandial period, probably due to the increase in uric acid.

In a study performed with samples of human subcutaneous abdominal adipose depots, C. Carpéné and coworkers illustrated that some phenolic compounds did not clearly block amine oxidases (AO) activities, whereas they impaired the interaction between hydrogen peroxide and the chromogenic mixture in fluorometric assays, which could be misinterpreted as an AO inhibition. Only resveratrol and quercetin partially impaired monoamine oxidases- (MAO-) dependent [^14^C]-tyramine oxidation, whereas measurements with the radiochemical method using [^14^C]-benzylamine clearly indicated that resveratrol did not inhibit human semicarbazide-sensitive AO (SSAO). Therefore, when measuring MAO or SSAO inhibition by antioxidants, the verification of the putative interaction of these agents with hydrogen peroxide or chromogenic mixture should carefully accompany testing.

The manuscripts in this special issue show that caution must be taken when interpreting human, animal, or* in vitro* studies that evaluate the effects of natural functional compounds in relation to oxidative stress. Both measurement and interpretation are far from straightforward and there is a desperate need for standardization, especially since decades of research still leave a murky picture without clear definites regarding the bioremedial effects of supplemented antioxidants. This significantly hinders clinical trials and introduction of such compounds in a routine clinical setting. Nonetheless, we sincerely hope that this special issue contributes to shedding some light on the meaning of oxidative stress markers and the bioremedial capacity of natural functional components in experimental models and human studies.

